# Fluorescence Lifetime Multiplexing with Environment‐Sensitive Chemigenetic Probes

**DOI:** 10.1002/cbic.202500174

**Published:** 2025-05-16

**Authors:** Sarah Emmert, Anna Rovira, Pablo Rivera‐Fuentes

**Affiliations:** ^1^ Department of Chemistry University of Zurich 8057 Zurich Switzerland

**Keywords:** cell imaging, chemigenetic probes, fluorescence, fluorescence lifetime imaging, HaloTag, protein structure prediction

## Abstract

HaloTag (HT) is a versatile self‐labeling protein that has been widely adopted in fluorescence microscopy. Besides its established use as an intensity‐based marker and sensor, interest in using HT for multiplexed fluorescence lifetime imaging microscopy (FLIM) has recently arisen. Herein, the application of an environment‐sensitive fluorophore was explored for FLIM multiplexing with the free dye and HT mutants. The extended coumarin pyridinium scaffold was selected due to its structural simplicity and the strong sensitivity of its photophysical properties to the environment. It was demonstrated that three‐channel imaging is possible by taking advantage of the propensity of the dye to accumulate in mitochondria and vesicles and the efficient labeling of two distinct HT mutants. Further investigation was conducted on different dehalogenase proteins and their FLIM multiplexing capabilities when paired with an HT mutant. Finally, the polarity of the protein binding pocket was identified as a key feature that affects the lifetime of this kind of fluorescent molecule.

## Graphical abstract

The fluorescence lifetimes of coumarin‐pyridinium dyes are sensitive to the environment, with lifetimes decreasing as the polarity of the solvent increases. Herein, it is shown that this trend is preserved in the binding pockets of proteins, enabling multiplexed imaging of three targets in live cells with a single fluorophore, and informing the design of chemigenetic tools for fluorescence lifetime imaging.

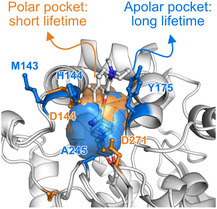

## 1 Introduction

Fluorescence microscopy allows us to observe living specimens in a noninvasive manner and is therefore one of the most powerful platforms to study highly dynamic biological processes. However, the amount of information obtained in an experiment is limited by the number of signals that can be measured and distinguished in the same sample, which typically depends on the spectral separation of the employed fluorophores. One way to increase the number of available channels is by fluorescence lifetime imaging microscopy (FLIM).^[^ ^1^, ^2^ ^]^ The additional temporal dimension enables highly multiplexed imaging with the available channels resulting from a combination of separable spectral and lifetime signals. Recently, the self‐labeling protein HaloTag (HT) was engineered for application in FLIM multiplexing.^[^ ^3^ ^]^ Judicious mutation of HT yielded several variants that influenced the lifetime of the bound small‐molecule fluorophores to achieve separable signals. Up to three targets could be labeled with the same fluorophore, and the signals could be discriminated through lifetime unmixing.^[^ ^3^ ^]^

**Figure 1:**
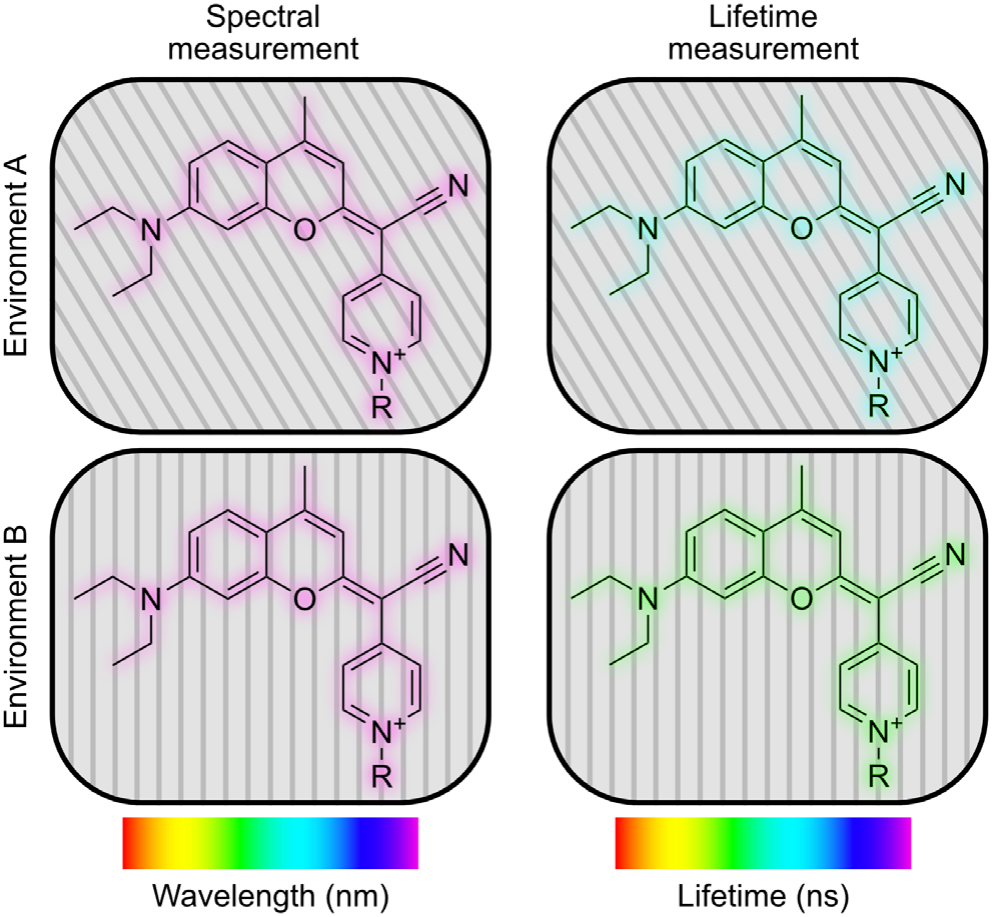
Environment‐sensitive coumarin pyridinium dyes exhibit similar spectral properties but varying fluorescence lifetime in different environments.

Xanthene‐based dyes are the fluorophores of choice for chemigenetic imaging with HT.^[^ ^4^, ^5^ ^]^ Notwithstanding their excellent brightness, photostability, and fluorogenicity, other fluorophores feature more dramatic environment‐dependent changes in their photophysical properties and are hence particularly interesting for testing the possibilities of FLIM multiplexing.^[^ ^6^ ^]^ Among these dyes are the coumarin pyridinium (COUPY) chromophores (**Figure** **1**), which are simple to synthesize and present favorable photophysical properties for bioimaging applications, including emission in the far‐red region and large Stokes’ shifts.^[^ ^7^ ^]^ We hypothesized that binding to a self‐labeling protein like HT would change the environment of a COUPY dye significantly, manifesting in a large shift in fluorescence lifetime. Furthermore, we envisioned that even small changes in the HT protein, close to the binding site of the molecule, could lead to distinguishable differences in lifetime. With the inherent environment‐sensitivity of the COUPY dyes, a small screening effort, rather than an extensive directed evolution campaign, should yield appropriate mutants for multiplexing. Hence, the goal of this work is not to develop the brightest and most photostable probes for bioimaging, but rather to study the effect of small but specific environmental changes in highly polarizable dyes and the applicability of this concept to multiplexed FLIM.

## 2 Results and Discussion

HT was specifically evolved to bind to and induce fluorescence turn‐on in haloalkane‐functionalized rhodamine dyes.^[^ ^8^ ^]^ Other ligands are bound less efficiently^[^ ^9^ ^]^ or the fluorescence enhancement is much weaker,^[^ ^4^, ^10^ ^]^ and therefore some engineering of the protein is required to optimize the interaction. For example, an HT variant was engineered specifically for styrylpyridinium dyes.^[^ ^11^ ^]^ Because the structure of COUPY dyes is reminiscent of those, we suspected that some of the mutants identified in that study could also have interactions with COUPY dyes, leading to changes in their fluorescence lifetime.

**Figure 2:**
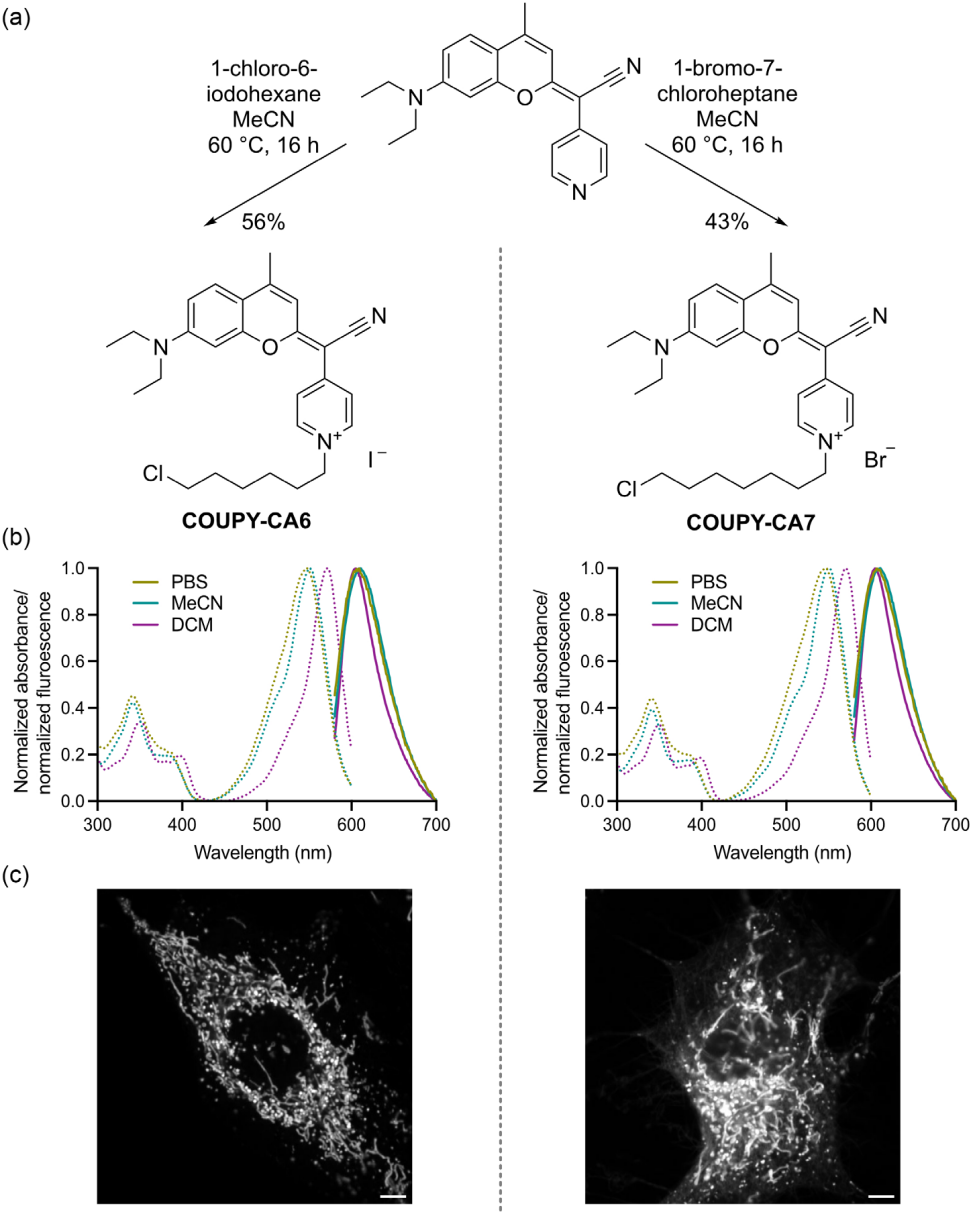
Synthesis of coumarin pyridinium‐based HaloTag ligands a), their absorbance and emission spectra b), and their respective localization in living HeLa cells c). Non‐transfected cells were incubated with 100 nM of the respective dye for 4 h. Scale bars = 5 μm.

The structure of COUPY dyes can be easily adapted with an HT‐binding chloroalkane (CA) chain during *N*‐alkylation of the pyridinium moiety. As the length of the CA chain has previously been proven relevant for binding efficiency,^[^ ^10^ ^]^ we synthesized two derivatives with a C6 (COUPY‐CA6) and C7 linker (COUPY‐CA7), respectively (**Figure** **2**a). Both fluorophores had almost identical absorbance and emission spectra and exhibited the desired environment‐sensitivity of the photophysical properties (Figure 2b, **Table** **1**). As the COUPY dyes are not fluorogenic, we first measured their signal in living HeLa cells without any HT being expressed. Both compounds seem to have an intrinsic tendency to accumulate in mitochondria, with some signal also in vesicles (Figure 2—Pearson's correlation coefficient with MitoTracker DeepRed = 0.7 ± 0.1), probably due to their permanent positive charge. However, with COUPY‐CA7, some weaker signals from other parts of the cell are also visible, presumably the endoplasmic reticulum (ER, Figure 2c). Therefore, we decided to proceed only with COUPY‐CA6, since for lifetime unmixing experiments, clearly localized signals are preferable.

**Table 1:** Photophysical properties of COUPY‐CA6 in different solvents and bound to two mutants of the HaloTag (HT) protein. Mutant acronyms are defined in Table S1.

Environment	λ_ex,max_ (nm)	λ_em,max_ (nm)	Φ_F_	τ (ns)
CH_2_Cl_2_	572	604	0.70	5.2a)
MeCN	550	610	0.19	1.8a)
PBS	547	609	0.11	1.4a)
HT‐CMHYA in PBS	573	615	0.73	5.9b)
HT‐MHYAD in PBS	571	612	0.52	5.5b)

a) Lifetimes of COUPY‐CA6 in different solvents were measured in cuvettes by time‐correlated single‐photon counting.

b) Lifetimes of COUPY‐CA6 bound to HT mutants were measured in cells by fluorescence lifetime imaging microscopy.

Since the reliability of lifetime unmixing increases with the photon count,^[^ ^12^ ^]^ we wanted to identify HT mutants that yield a bright signal when conjugated to COUPY‐CA6. For our screening, we selected 10 mutants that were previously identified to affect the fluorogenicity of the styrylpyridinium dye.^[^ ^11^ ^]^ These mutants included the most widely used HT7, the five best hits for the styrylpyridinium dye, and mutants that introduced additional charge. All mutant proteins were conjugated to COUPY‐CA6, and their brightness and fluorescence lifetime were measured in vitro (Table S1, Supporting Information). We found several mutants that were 2 to 5‐fold brighter than HT7: HT‐CMHYA, HT‐CYA, HT‐MHYAD, HT‐YAD, and HT‐YD (mutant acronyms are defined in Table S1, Supporting Information). The observed lifetimes ranged from 4.9 to 5.9 ns, which leads to a difference in lifetime of more than 3 ns compared to the unbound dye in cells (1.4 ns).

**Figure 3:**
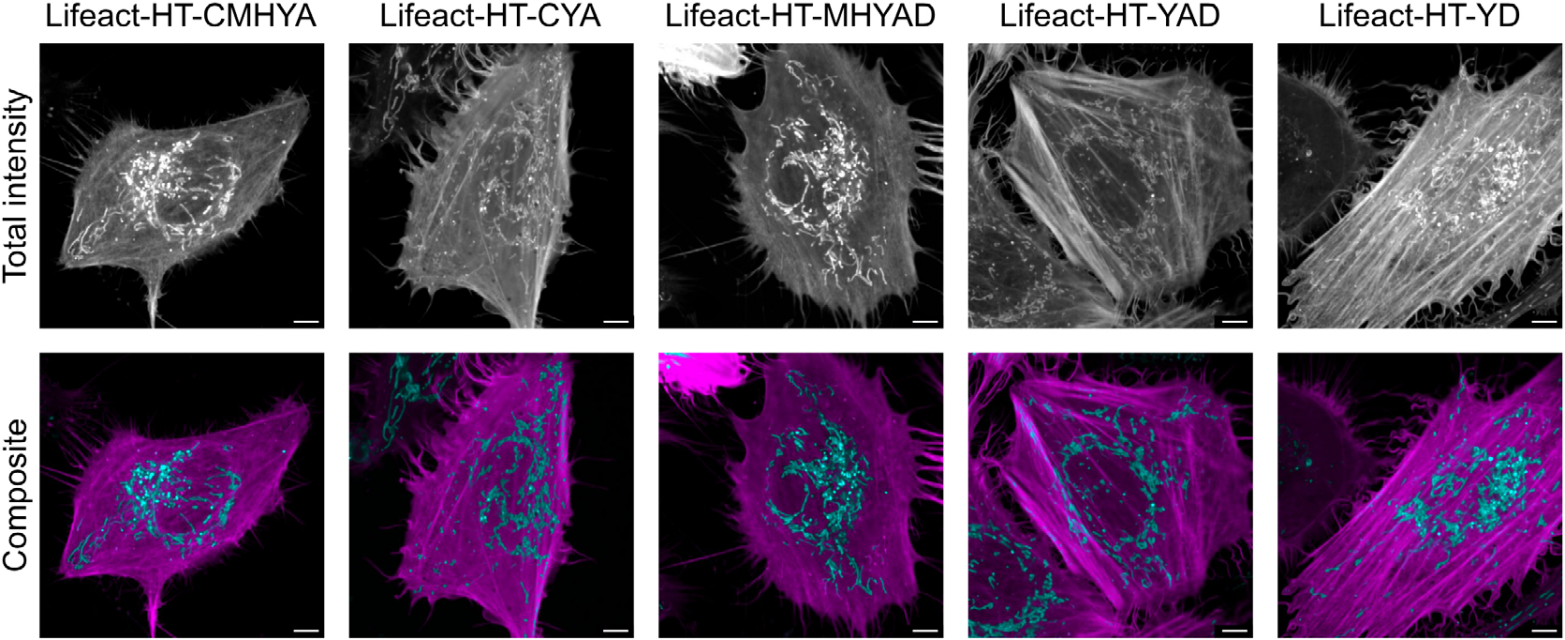
Fluorescence lifetime imaging microscopy of HeLa cells expressing various HaloTag versions fused to actin and incubated with 500 nM COUPY‐CA6 for 16 h. For each transfection condition, the total intensity and a composite image of lifetime‐unmixed channels are depicted. Lifetime unmixing was performed with the phasor method. Phasor plots are displayed in Figure S3. Lifeact: actin targeting. Scale bars = 5 μm.

Based on the large difference in lifetime between the free COUPY dye and the HT conjugates, we hypothesized that these signals could be separated by FLIM. The HT variants were expressed fused to actin in live HeLa cells and labeled with COUPY‐CA6. Indeed, the signal from the unbound and bound dye could be distinguished by lifetime unmixing (**Figure** **3**). Lifetime unmixing enabled a clean signal from the labeled structure even though the COUPY‐CA6 is non‐fluorogenic and accumulates in mitochondria and vesicles. The signal from the unbound dye can be easily separated by the phasor method.^[^ ^13^ ^]^

Subcellular compartments have their own environment, often varying dramatically in pH, redox potential, protein content, etc. We therefore asked whether the specific cellular environment or the HT mutant had a larger influence on the fluorescence lifetime. We prepared plasmid constructs to express either the same or different HT mutants in the Golgi apparatus (Galt1) and the nucleus (H2B). These compartments have little spatial overlap and can be captured well in the same z‐plane. These characteristics facilitate the comparison of unmixing results. Considering the variation in fluorescence lifetime observed in vitro (Table 1 and Table S1, Supporting Information), we expected good signal separation for the variants HT‐CMHYA and HT‐MHYAD.

**Figure 4:**
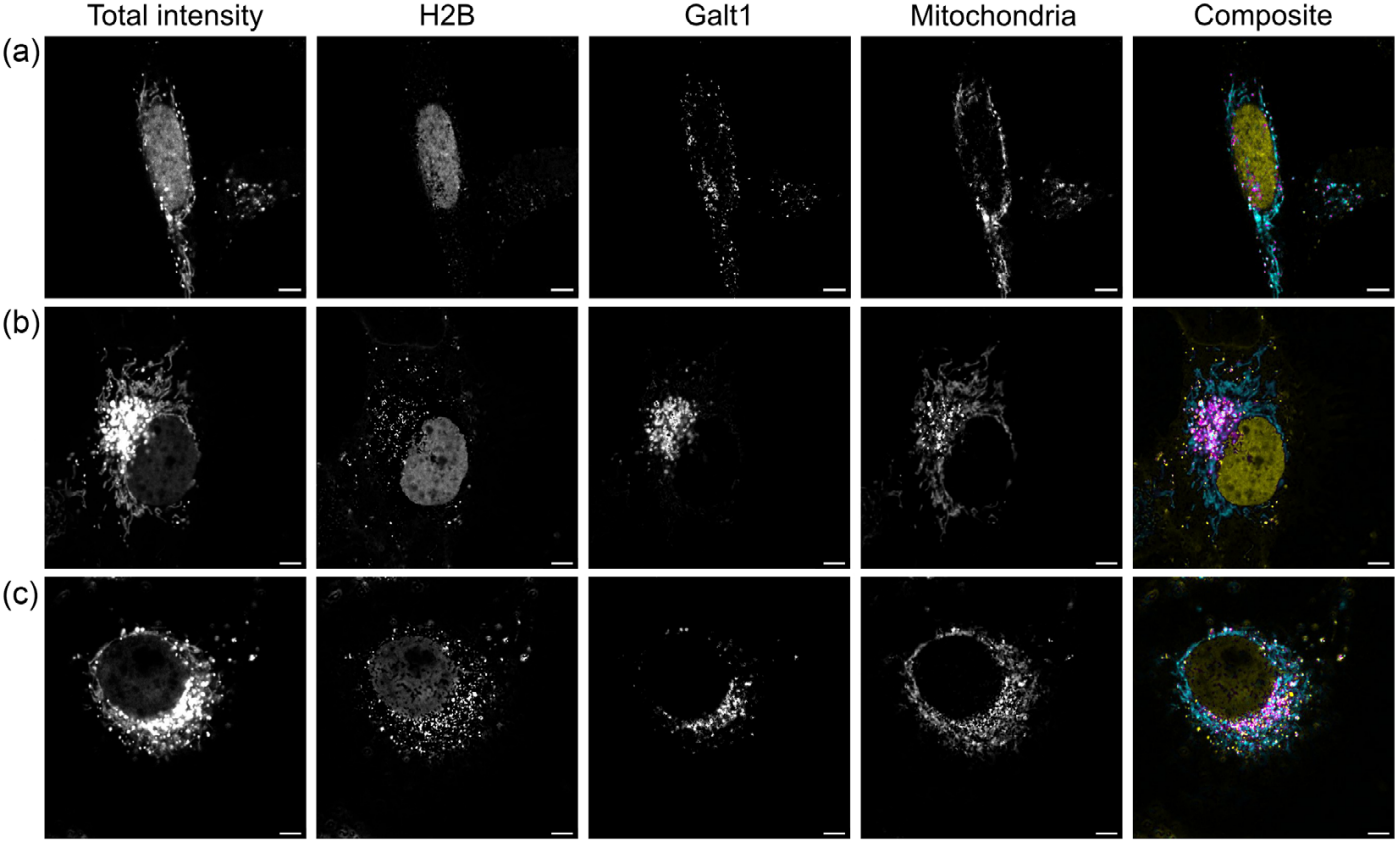
Fluorescence lifetime imaging microscopy of HeLa cells expressing Galt1(Golgi)‐HT‐CMHYA + H2B(nucleus)‐HT‐CMHYA a), Galt1(Golgi)‐HT‐CMHYA + H2B(nucleus)‐HT‐MHYAD b), or Galt1(Golgi)‐HT‐CMHYA + H2B(nucleus)‐DppA‐mGold c) and incubated with COUPY‐CA6. For each transfection condition, the total intensity, the separated species, and a composite image are depicted. Lifetime unmixing was performed with the phasor method. Phasor plots are displayed in Figure S3. Scale bars = 5 μm.

Both the subcellular environment and the HT variant seem to influence the lifetime of the fluorophore (**Figure** **4**a,b). Different HT variants clearly lead to better unmixing results, especially because several combinations can be tested for a given set of subcellular localizations. However, as both effects act together, the combination of variants should be optimized for each set of subcellular compartments. Moreover, it was important to optimize the plasmid concentration and the plasmid ratios for co‐transfection (Table S2, Supporting Information) as more similar signal intensities led to fewer artifacts in the single channels after unmixing. The concentration of COUPY‐CA6 for incubation and the wash time were utilized to adjust the brightness of the mitochondrial and vesicular signal originating from the unbound dye.

A recent study on the optimization of HT labeling pointed out the potential of also considering other dehalogenase enzymes for non‐rhodamine ligands.^[^ ^14^ ^]^ Inspired by this work, we assessed whether better lifetime unmixing could be achieved by using entirely different proteins rather than mutants of HT. We chose six alternative dehalogenases, DbeA, DbjA, DhlA‐W175Y, DmmA, DppA, and LinB.^[^ ^14^ ^]^ In line with HT engineering, we introduced a His → Phe mutation in the active site of each dehalogenase to enable covalent labeling. Only one of the proteins, DppA, gave a measurable signal in HeLa cells after incubation with COUPY‐CA6 (Figure S1). We prepared a plasmid construct with DppA targeting the nucleus as a fusion to the fluorescent protein mTurquoise2.^[^ ^15^ ^]^ mTurquoise2 allowed us to identify transfected cells easily despite the dim signal of the dehalogenase‐bound COUPY‐CA6. We expressed this construct together with Galt1(Golgi)‐HT‐CMHYA in HeLa cells and incubated with the dye. Good unmixing of the three labeled structures was observed even though the nuclear signal exhibits some artifacts as bright spots (Figure 4c). We envisioned that further engineering of the DppA protein could lead to brighter conjugates with a different fluorescence lifetime.

**Figure 5:**
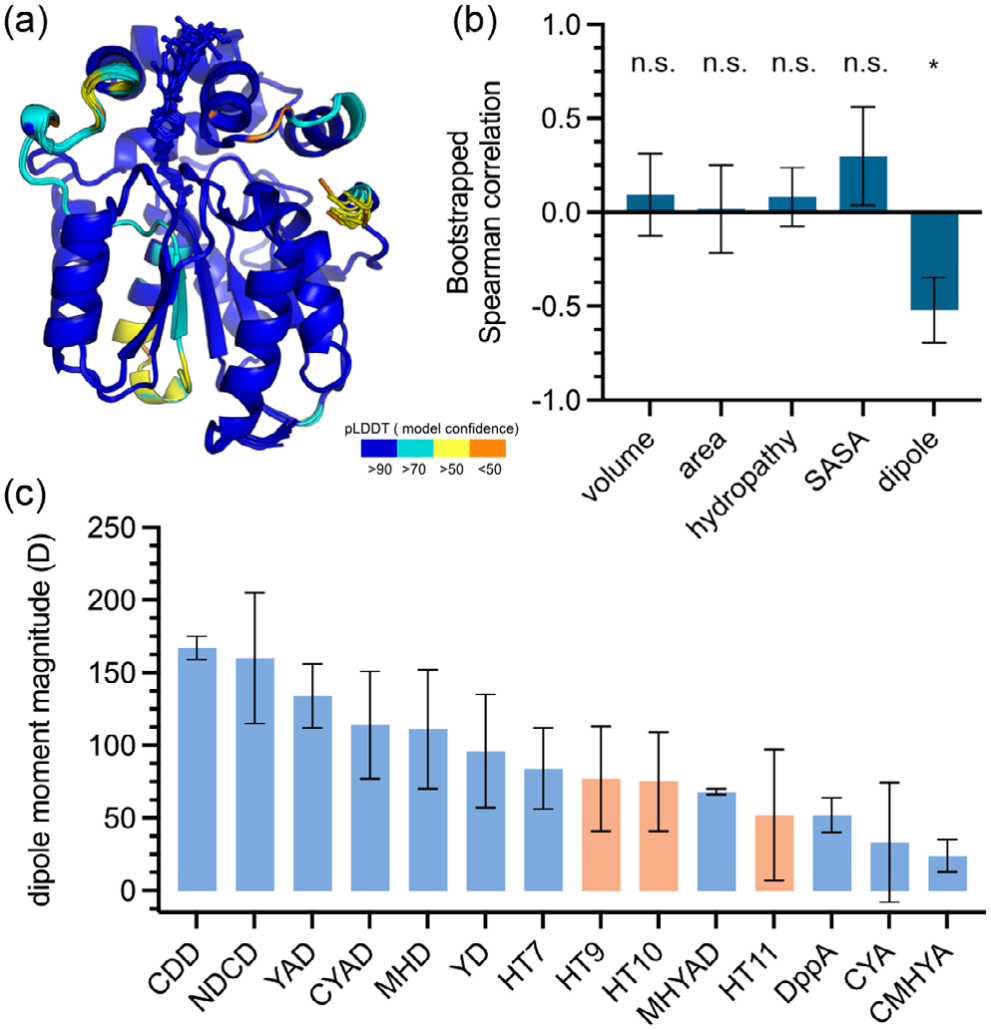
Binding pocket properties and correlation with experimental fluorescence lifetimes. a) Overlay of the structures of all mutants of HaloTag calculated with Boltz‐1. Both the protein (cartoon) and the ligand (spheres and sticks) are colored by pLDDT. b) Correlation coefficients between experimentally observed fluorescence lifetime and calculated properties of the binding pocket of the proteins. Significance levels were assessed by a bootstrapping confidence interval (Supporting Information). n.s. = not significantly different from 0 within a 95% confidence interval. *Significantly different from 0 within the 95% confidence interval. c) Calculated magnitudes of the dipole moment of the pocket for all proteins reported in this study.

Finally, to gain some insight into specific properties of the protein that dictate the observed fluorescence lifetimes of COUPY‐C6, we modeled all HT mutants discussed here and the DppA dehalogenase using Boltz‐1,^[^ ^16^ ^]^ an all‐atom biomolecular modeling tool akin to AlphaFold3.^[^ ^17^ ^]^ All proteins were predicted with very high global predicted local distance difference test (pLDDT, **Figure** **5**a). We computed 10 models per protein to account for variability in the predicted structures. The COUPY‐C6 ligand was predicted to occupy virtually the same position in all proteins and models (except for DppA, see Figure S5). Because all HT mutants displayed very similar binding pockets, we calculated the volume, surface area, hydropathy,^[^ ^18^ ^]^ solvent‐accessible surface area,^[^ ^19^ ^]^ and the dipole moment of the binding pocket defined by all amino acids within a 5 Å of the dye (for details, see the Supporting Information). We calculated the Spearman correlation coefficient between these properties and the observed lifetime for all HT mutants, which revealed that the magnitude of the pocket's dipole moment is moderately anticorrelated (ρ = −0.521 ± 0.174) with the fluorescence lifetime. No other properties displayed a significant correlation (Figure 5b). Notwithstanding the limitations of this analysis, these results suggest that the polarity of the binding pocket has the strongest effect on the lifetime of the fluorophore. This conclusion is consistent with the trend observed in solution, where the lifetime of COUPY dyes varies dramatically with the polarity of the solvent.^[^ ^7^ ^]^ Of note, HT mutants, HT9, HT10, and HT11, were reported to change the fluorescence lifetime of rhodamine‐based dyes. Calculation of their pocket dipole moments reveals that HT11 has the least polar pocket (Figure 5c), which could lead to long lifetimes for COUPY dyes. This mutant, however, has a tryptophane at position 175 that quenches the excited state of rhodamine dyes, leading to a decrease in the fluorescence lifetime. This quenching, which would likely extend to COUPY dyes given their proximity in our models (Figure S5), would counteract the effect of the apolar binding pocket. It is difficult to predict which effect—pocket polarity or tryptophane quenching—would have a larger effect on the observed lifetime. A systematic study in which the distance between the fluorophore and tryptophane residues is varied and the dipole moment is calculated could answer this question.

## 3 Conclusion

The environment‐sensitive COUPY‐CA6 changes its lifetime when bound to mutants of the self‐labeling protein HT. This characteristic can be exploited for FLIM multiplexing. We found two scenarios that could be exploited for multiplexing: a) The same mutant can display different lifetimes in different organelles, and b) different mutants can give different lifetimes. The former is a key result of this study and is worth exploring also for other dye classes (e.g., rhodamine‐based probes). Additionally, we showed that unbound dye can give a third, separable signal to label an additional location. These results were obtained by screening a small library of only 10 variants, demonstrating that COUPY dyes have a pronounced sensitivity to their environment. In addition to using variants of the same protein, different dehalogenase proteins lead to separable signals by FLIM. We demonstrated this hypothesis using the DppA protein, despite its weak fluorescent signal with COUPY‐CA6. We envision that the brightness could be increased for DppA by directed evolution, similar to how HT was developed. Such efforts could lead to more robust chromophore‐protein pairs that are lifetime‐orthogonal to HT‐based probes.

Biomolecular modeling using Boltz‐1 revealed correlation between the magnitude of the pocket's dipole moment and the observed lifetime. This correlation mirrors the behavior of COUPY and other dyes in solution. We foresee, however, that this correlation would be weaker for dyes that do not bind to cavities, but rather to the surface of the protein (e.g., rhodamine dyes). Nevertheless, the dipole moment of the local environment of the probe could be used to guide the design of new mutants to engineer chemigenetic probes with tailored fluorescence lifetimes.

Additionally, because not only shifts in fluorescence lifetime but also spectral shifts are observed for COUPY‐CA6 in solution and bound to HT (Figure S4), we posit that further engineered dehalogenases could enable more complex imaging modalities like spectrally‐resolved FLIM^[^ ^2^ ^]^ to further improve unmixing. As we stated before, COUPY dyes are not fluorogenic, and the mitochondrial/vesicular signal of the unbound dye could be a disadvantage in many experiments. Thus, finding a dye that is both fluorogenic and has a wide range of fluorescence lifetimes is an interesting challenge and could unlock new possibilities for multiplexed imaging.

## Conflict of Interest

The authors declare no conflicts of interest.

## Supporting information

Supplementary Material

## Data Availability

Data supporting this work are available in the supporting information and through Zenodo (DOI: 10.5281/zenodo.14929463). Code supporting this paper is available at https://gitlab.uzh.ch/locbp/public/lifetime_protein_pocket.
