# Supplementary material for: Fluorescence Lifetime Multiplexing with Environment‐Sensitive Chemigenetic Probes
Source: Chembiochem. 2025 May 16;26(12):e202500174. doi: 10.1002/cbic.202500174 (PMC12177688; doi:10.1002/cbic.202500174)
Supplement: Supplementary file 1 — Supplementary Material [file CBIC-26-e202500174-s001.pdf]

Fluorescence Lifetime Multiplexing with Environment-Sensitive Chemigenetic Probes

Supporting Information

Sarah Emmert,[1,2] Anna Rovira[1,2] and Pablo Rivera-Fuentes[1*]

1) Department of Chemistry, University of Zurich, Zurich, Switzerland.
2) These authors contributed equally.

*Correspondence to: pablo.riverafuentes@uzh.ch

Table of contents

1 General experimental methods 2
2 Experimental details 2
3 Supplementary Figures 5
4 Supplementary Tables 8
5 Protein pocket analysis 11
6 Synthetic procedures 12
7 NMR spectra 14
8 References 16

1 General experimental methods

Unless stated otherwise, all reagents and solvents were purchased from commercial sources and used as received. NMR spectra were acquired on Bruker AVANCE NEO-400 and Bruker AVANCE III-400 instruments. ^{1}H NMR chemical shifts are reported in ppm relative to $SiMe_4$ (δ = 0) and were referenced internally with respect to residual protons in the solvent (δ = 7.26 for $CDCl_3$)[1]. Coupling constants are reported in Hz. ^{13}C NMR chemical shifts are reported in ppm relative to $SiMe_4$ (δ = 0) and were referenced internally with respect to solvent signal (δ = 77.16 for $CDCl_3$).[1] High-resolution mass spectrometry (HRMS) was performed by the MS facility of UZH. Reaction progress was followed by thin-layer chromatography (TLC). Purification by flash column chromatography was performed using a Biotage® Selekt system and Büchi FlashPure columns. IUPAC names of all compounds are provided and were determined using CS ChemDraw 19.1.

2 Experimental details

Optical spectroscopic methods

Stock solutions were prepared in DMSO (spectrophotometric grade >99.9%) at concentrations of 50 mM and stored at -20 °C. UV-Vis spectra were acquired using a Multiskan SkyHigh Microplate Spectrophotometer (ThermoFisher Scientific) and quartz cuvettes using SkanIt software. Fluorescence spectra were acquired using an FS5 Spectrofluorometer (Edinburgh Instruments) equipped with an SC-40 plate reader and quartz cuvettes using Fluoracle software. The obtained spectra were background corrected. Quantum yields were measured at concentrations of 5 µM using an SC-30 integrating sphere and quartz cuvettes. Lifetimes in solution were acquired using a Hamamatsu Quantaurus-Tau Fluorescence Lifetime Spectrometer (C11367-31), which is equipped with a photon counting measurement system. The excitation wavelength was set at 365 nm. A 1 component fit was applied to the obtained data. All measurements were carried out as three technical replicates and at 20 °C unless stated otherwise. Mean values of three technical replicates are given in all tables.

Co-localization analysis.

HeLa cells (passage 7) were plated at a density of 30,000 cells per well in an Ibidi 8-well plate 24 h before observation. Cells were treated with 0.1 µM COUPY-C6 and 0.1 µM MitoTracker DeepRed® for 30 min at 37 °C. After two washing steps with PBS, FluroBrite medium was added. Imaging was carried out using a spinning disk confocal microscope (Nikon). COUPY-C6 was imaged using an excitation laser at 561 nm and

and emission filter at 600/52, whereas MitoTracker DeepRed® was excited at 640 nm and emission was collected in the 708/75 window. 10 fields of view were collected, and each one was analyzed with custom Python code to calculate the all-pixel (no background exclusions) Pearson's correlation coefficient. The mean and standard deviation of the 10 fields of view gave a Pearson's correlation coefficient of 0.7±0.1.

Fluorescence lifetime imaging microscopy (FLIM)

FLIM was performed with a Leica SP8 FALCON equipped with a white laser (561 nm selected) and a HyD detector in photon counting mode (580–700 nm). Images were collected using an HC PL APO 63x/1.40 Oil CS2 objective. The instrument was operated using the LAS X Navigator software. Imaging was performed at 37 ºC in a 5% CO_2 environment. Images were analyzed with the LAS X Falcon Phasor software by applying a wavelet filter[2] and positioning the cluster circles at the edge of each species in the phasor plot. Final images were visualized with Fiji (ImageJ).

Cloning

Primers for Gibson assembly were designed using SnapGene. Respective precursor plasmids and primers are reported in Table **S1** and Table **S3**. Reagents and general procedures from the Gibson Assembly Cloning Kit from New England Biolabs (NEB) were used. Vector and insert fragments were linearized by polymerase chain reaction (PCR) and template DNA was digested with DpnI. PCR fragments were purified with the QIAquick PCR Purification Kit from Qiagen. Backbone and insert fragment were ligated and transformed by heat shock into DH5α competent cells following the provided standard procedure of the vendor. Plasmids were amplified by incubation of lysogeny broth (LB) cultures containing the appropriate antibiotics overnight at 37 °C. DNA was isolated using the Qiagen Plasmid Mini Kit or the Qiagen Plasmid Plus Midi Kit. The correct sequence of the gene of interest (GOI) was confirmed by the Sanger sequencing service of Microsynth.

Site-directed mutagenesis

Primers for site-directed mutagenesis were designed with NEBaseChanger. Respective precursor plasmids and primers are reported in Table **S1** and Table **S3**. The standard procedure and reagents from the Q5 Site-Directed Mutagenesis Kit (NEB) were used to insert mutations, ligate plasmids and transform them into DH5α competent cells by heat shock. The correct sequence of the GOI was confirmed by the Sanger sequencing service of Microsynth.

Protein expression

BL21(DE3) competent cells from NEB were transformed with the respective plasmid by heat shock following the provided standard procedure of the vendor. A single colony was inoculated into LB medium containing the appropriate antibiotics and incubated at 37 °C overnight. From the starter culture fresh medium was inoculated and incubated at 37 °C until OD=0.4–0.8 was reached. Isopropyl β-D-1-thiogalactopyranoside (IPTG) was added to a final concentration of 1 mM and the culture was further incubated at 18 °C overnight. *E. coli* were harvested by centrifugation and resuspended in HEPES buffer (20 mM HEPES, 300 mM NaCl, pH=7.4). Glycerol was added to a final concentration of 10% as well as Turbonuclease (5 µL) and a protease inhibitor cocktail tablet (Roche). The cells were lysed by sonication (70% amplitude,10 s pulse/10 s pulse off for 2.5 min). The lysate was cleared by centrifugation and the protein was purified by Ni-His-affinity chromatography in batch-mode. Fractions were analyzed by gel electrophoresis and pure fractions were pooled and dialyzed against PBS.

Cell culture

HeLa cells were grown in Dulbecco's Modified Eagle Medium (DMEM) supplemented with fetal bovine serum (FBS, 10%) and penicillin (100 U mL^{-1})/streptomycin (100 µg mL^{-1})/fungizone (0.25 µg mL^{-1}) at 37 ºC in 5% CO_2 environment. For imaging, 15'000–20'000 cells were seeded per well of an 8-well Ibidi chambered cover glass 2–3 days prior to imaging. If required, cells were transfected with plasmid DNA using jetPRIME according to the recommended protocol of the supplier 1–2 days prior to imaging. The cells were incubated with the respective probes in growth medium for the indicated time. Before imaging, the growth medium was removed, the cells were washed with PBS (2x) and imaged in FluoroBrite DMEM.

3 Supplementary Figures

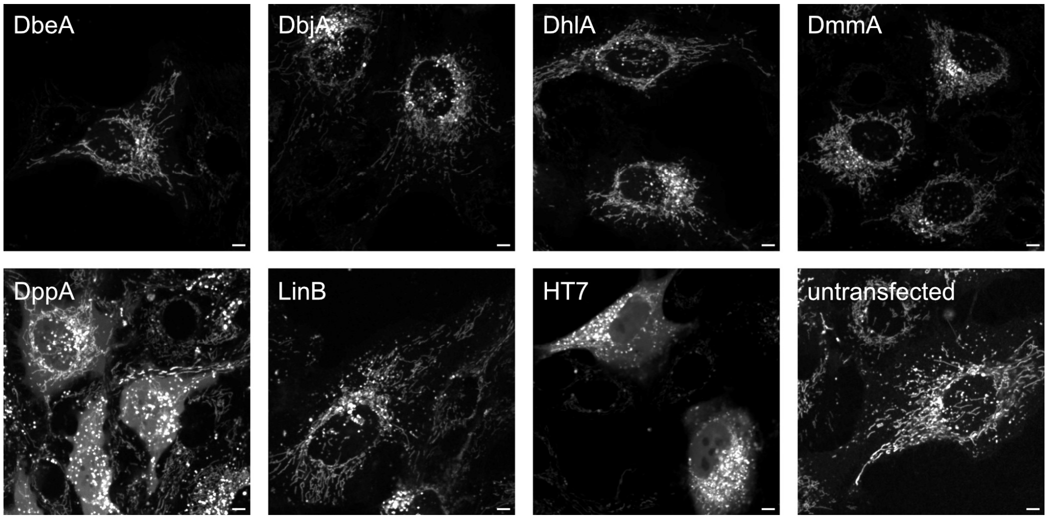

Figure S1. HeLa cells transfected with various dehalogenases and stained with 0.5 µM COUPY-CA6 for 16 h (washed 3x 1 h with fresh growth medium). Confocal imaging was performed on a Nikon W1 spinning disc microscope equipped with an sCMOS camera (Photometrix) and a CFI Plan Apochromat Lambda D oil immersion objective (60x, NA = 1.4). Excitation laser 561 nm, emission filter 600/52 nm. The microscope was operated using the NIS elements software. Imaging was performed at 37 ºC in a 5% CO_2 environment. Images were visualized in Fiji (ImageJ).

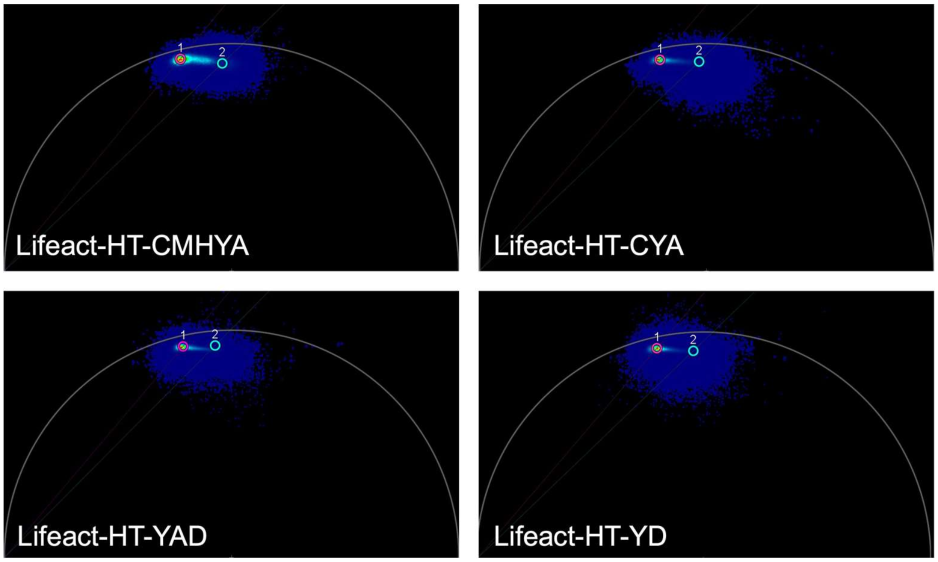

Figure S2. Phasor plots for the images in Figure 3.

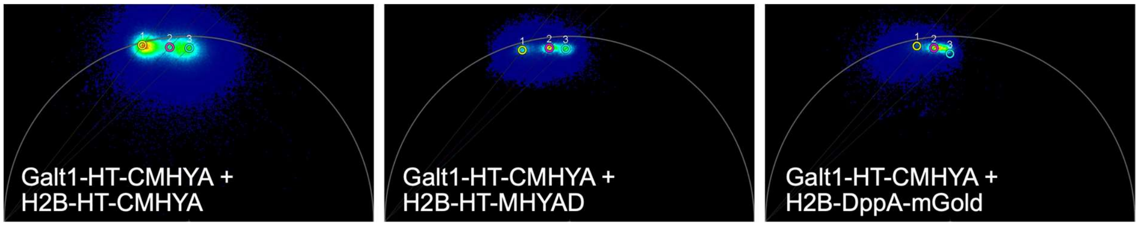

Figure S3. Phasor plots for the images in Figure 4.

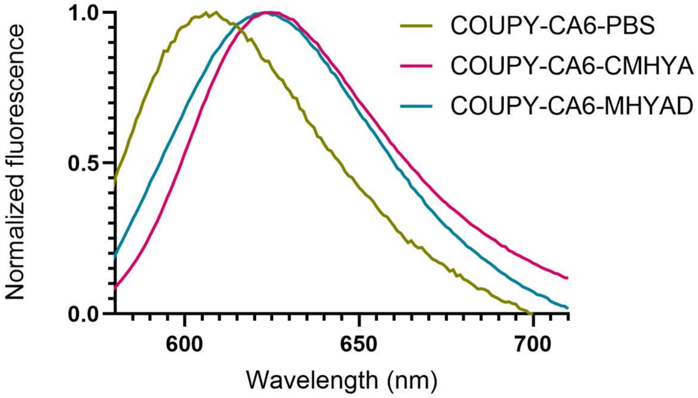

Figure S4. Comparison of the fluorescence spectra of COUPY-CA6 in PBS and bound to HT-CMHYA and HT-MHYAD.

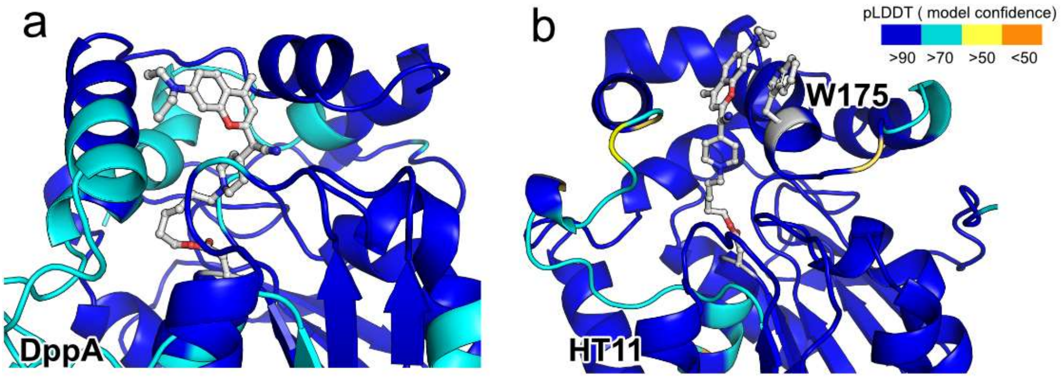

Figure S5. Boltz-1 models of COUPY-C6 bound to DppA and HT11. a) DppA has a shallower binding pocket than HT7, thus the linker in COUPY-C6 seems to be too long and is “contorted” in the binding pocket. As a consequence, it is likely that in reality, the dye reaches out into the solvent much more than in HT mutants, thus leading to the significantly shorter lifetime. For this reason, the DppA models were not used for correlation analysis. b) HT11 mutant with W175 highlighted in white. The proximity to the COUPY dye would likely result in fluorescence quenching and lifetime shortening.

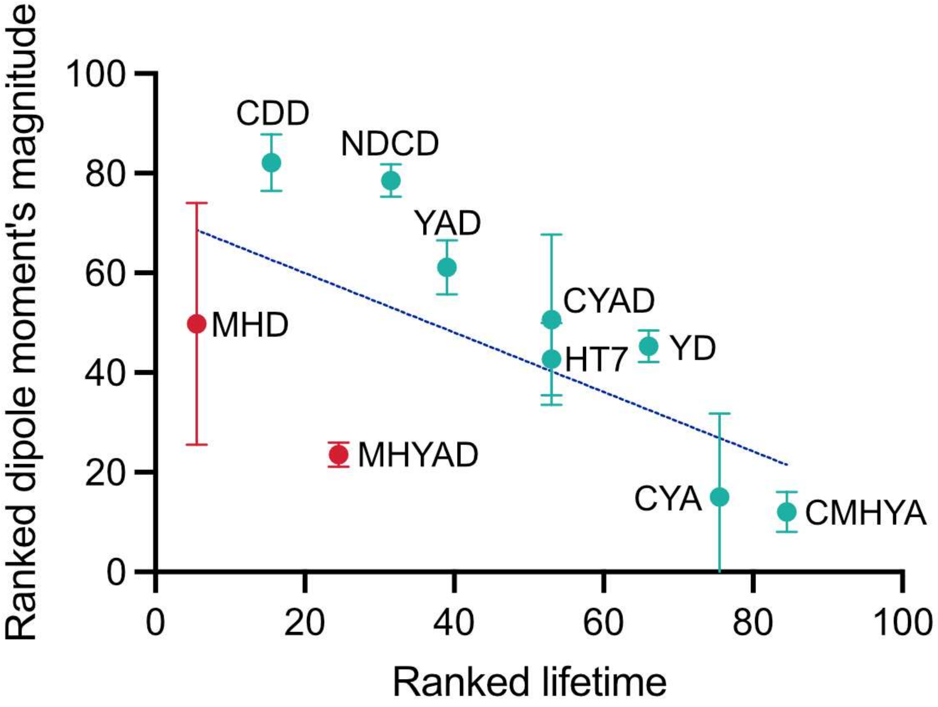

Figure S6. Plot of ranked lifetime and dipole moment's magnitudes for the proteins used in Spearman correlation analysis. The plot reveals that mutants MHD and MHYAD do not follow the trend of the rest of the proteins. Dots are means and whiskers standard deviation. The dotted line represents the best linear fit including all proteins displayed on the graph.

4 Supplementary Tables

Table S1. Screening HT mutant proteins conjugated to COUPY-CA6 for brightness and fluorescence lifetime. In a 96-well plate, solutions of the HT variants (4 µM) and COUPY-CA6 (2 µM) in PBS were prepared and FLIM was performed. For the whole field of view the lifetimes were analyzed (2 components fit to account for residual unbound dye). The intensity sum was used to compare the brightness of mutants. The relative brightness might also reflect the contribution of faster binding kinetics for some mutants. τ2 is used to compare the fluorescence lifetime (the longer of two lifetimes always had a higher contribution and likely corresponds to the bound species). Three technical replicates were measured.

Variant	Mutations	Brightness (relative)	τ (ns)
HT7-His6		1.0	5.6
HT-CDD-His6	R133C, F144D, L271D	0.5	5.3
HT-CMHYA-His6	R133C, E143M, F144H, M175Y, V245A	2.6	5.9
HT-CYA-His6	R133C, M175Y, V245A	3.4	5.8
HT-CYAD-His6	R133C, M175Y, V245A, L271D	0.8	5.6
HT-MHD-His6	E143M, F144H, L271D	0.3	4.9
HT-MHYAD-His6	E143M, F144H, M175Y, V245A, L271D	1.9	5.5
HT-NDCD-His6	R133N, F144D, M175C, L271D	0.7	5.6
HT-YAD-His6	M175Y, V245A, L271D	4.6	5.6
HT-YD-His6	M175Y, L271D	2.1	5.7

Table S2. Transfection and incubation conditions for FLIM multiplexing experiments.

Plasmid 1	Plasmid 2	COUPY-CA6 incubation	Wash
Galt1(Golgi)-HT-CMHYA (200 ng)	H2B(nucleus)-HT-CMHYA (100 ng)	100 nM for 3 h	overnight
Galt1(Golgi)-HT-CMHYA (200 ng)	H2B(nucleus)-HT-MHYAD (300 ng)	100 nM for 4 h	/
Galt1(Golgi)-HT-CMHYA (100 ng)	H2B(nucleus)-DppA-mGold (400 ng)	100 nM for 4 h	4 h

Table S3. Plasmid Sources.

Plasmid	Source	Vector precursor	Insert precursor
HT7-His6	[a]		
HT-CDD-His6	[a]		
HT-CMHYA-His6	[a]		
HT-CYA-His6	[a]		
HT-CYAD-His6	[a]		
HT-MHD-His6	[a]		
HT-MHYAD-His6	[a]		
HT-NCDD-His6	[a]		
HT-YAD-His6	[a]		
HT-YD-His6	[a]		
Lifeact-HT-CMHYA	[a]		
Lifeact-HT-CYA	[a]		
Lifeact-HT-MHYAD	[a]		
Lifeact-YAD	[a]		
Lifeact-HT-YD	[a]		
Galt1(Golgi)-HT-CMHYA	Gibson assembly	Galt1(Golgi)-HT-mGold	Lifeact-HT-CMHYA
H2B(nucleus)-HT-CMHYA	Gibson assembly	H2B(nucleus)-HT-mGold	Lifeact-HT-CMHYA
H2B(nucleus)-HT-MHYAD	Gibson assembly	H2B(nucleus)-HT-mGold	Lifeact-HT-MHYAD
DbeA-H280F	Gibson assembly	HT-mGold	[b]
DbjA-H280F	Gibson assembly	HT-mGold	[b]
DhlA-W175Y-H289F	Gibson assembly	HT-mGold	[b]
DmmA-H315F	Gibson assembly	HT-mGold	[b]
DppA-H278F	Gibson assembly	HT-mGold	[b]
LinB-H272F	Gibson assembly	HT-mGold	[b]
H2B(nucleus)-DppA-H278F-mGold	Gibson assembly		DppA-H278F

[a] Gift from Thomas Ward (University of Basel)
[b] Synthetic gene

Table S4. Primers for plasmid generation.

Plasmid	Vector forward	Vector reverse	Insert forward	Insert reverse
Galt1(Golgi)-HT-CMHYA	agatttcctaaagcggccg	aagccagtaccgatttcggt	aatcggtactggctttcca	ccgctttaggaaatctccagag
H2B(nucleus)-HT-CMHYA	accgctcgagagctaagcg	atttcggagccggtcgactg	gaccggctccgaaatcggt	tagctctcgagcggttatca
H2B(nucleus)-HT-MHYAD	accgctcgagagctaagcg	atttcggagccggtcgactg	gaccggctccgaaatcggt	tagctctcgagcggttatca
DbeA-H280F	ggaatgaggccgcgactct	tcggccatgagatctgagtccg	[c]	[c]
DbjA-H280F	cgtagattgacgcgactctaga	ttagacatggcggtctcgag	[c]	[c]
DhlA-W175Y-H289F	agagtgacgcgactctaga	ttaatcatggcggtctcgag	[c]	[c]
DmmA-H315F	cgagtgacgcgactctaga	cttgccatgagatctgagtccg	[c]	[c]
DppA-H278F	gcagtgacgcgactctaga	aactccatggcggtctcgag	[c]	[c]
LinB-H272F	ggcgtgacgcgactctaga	agactcatggcggtctcgag	[c]	[c]
H2B(nucleus)-DppA-H278F-mGold	tgggcaggagatttccggc	acgaactcgctgccggtcga	cggcagcgagttcgttaggac	gaaatctcctgcccaaacgc

[c] Insert overhangs were already included in the design of synthetic genes.

Table S5. Average fluorescence lifetimes of the used protein variants fused to actin, Galt1 and H2B. n.m.: not measured.

Protein	Actin	H2B	Galt1
YAD	4.82 ns	4.97 ns	4.29 ns
YD	4.77 ns	n.m.	n.m.
CYA	4.80 ns	n.m.	n.m.
CMHYA	4.92 ns	4.94 ns	4.33 ns
MHYAD	4.91 ns	5.05 ns	4.29 ns
DppA-mGold	n.m.	4.72 ns	n.m.

5 Protein pocket analysis

Boltz-1 modeling. To analyze the properties of the binding pockets of different HT mutants and DppA, we calculated the structure of the protein with the C6-COUPY dye bound covalently to D106 (for HT mutants) or D123 (for DppA) using the AlphaFold3 clone model Boltz-1.[3] Specifically, we used a new “restricted” version that preserves better the valence in ligands.[4] The calculation was run using the multiple sequence alignment server, with 25 recycles and 10 diffusion samples, which are the 10 models per protein that we used for statistical analysis downstream.

Model alignment. We wrote Python code to read the protein model files in CIF format from the `BoltzResults` folder. We defined model 0 from HT7 as the reference model (`alignment_reference.cif`) for the alignment of all other models and proteins. We used a temporary PyMOL script that loads both the reference CIF and the query CIF file, aligns the query model (using only CA atoms), and saves the aligned model in the appropriate output folder.

Calculation of Pocket Properties. The aligned CIF files were processed using Biopython’s MMCIFParser. The script identifies the modified residue (D106 for HT mutants or D123 or DppA) within the structure. This residue is split into two parts: one is re-assigned as a standard aspartate and the other as a ligand. The code collects ligand atoms from the LIG residues and computes their centroid. It then identifies pocket residues as those whose atoms are within a user-defined distance cutoff from the ligand’s atoms.

Cavity Property Calculation. A temporary pocket PDB file is generated from the selected pocket residues. This file is passed to the PyKVFinder API[5] to calculate cavity properties such as volume, surface area, and hydropathy. The script also calculates the solvent accessible surface area (SASA) for the protein (excluding the ligand) using freesasa.[6] It also generates a PQR file via pdb2pqr,[7] which is parsed to compute the dipole moment of the pocket.

Downstream Analysis and Visualization. To understand which pocket properties correlate with the observed lifetime of the fluorophore, we performed a bootstrapped Spearman correlation analysis between the experimental lifetime and the various computed pocket properties. For each protein, one model is randomly sampled (with replacement) across 1000 iterations to generate distributions of Spearman correlation coefficients. The mean and standard deviation of these correlations are reported to assess the robustness of the relationships. Significance was assessed by calculating bootstrap percentile confidence intervals. We generated a bootstrap distribution of the correlation coefficient, then compute the lower (2.5th percentile) and upper (97.5th

percentile) bounds of this distribution. If the resulting 95% confidence interval does not contain 0, we considered that the correlation was significantly different from 0 at the 5% level.

The code and all input files, including Boltz-1 models, are available at https://gitlab.uzh.ch/locbp/public/lifetime_protein_pocket

6 Synthetic procedures

(*E*)-1-(6-Chlorohexyl)-4-(cyano(7-(diethylamino)-4-methyl-2*H*-chromen-2-ylidene)methyl)pyridin-1-ium iodide (COUPY-CA6)

1-chloro-6-iodohexane (183 µL, 1.21 mmol, 20 equiv.) was added to a solution of the coumarin (20 mg, 0.060 mmol, 1 equiv.) in anhydrous acetonitrile (2 mL). The solution was stirred for 16 h at 60 ºC. The residue was purified by flash column chromatography (SiO_2, 0 → 10 % MeOH in CH_2Cl_2) to yield a purple solid (24 mg, 68%). R_f (SiO_2, CH_2Cl_2/MeOH 90:10) = 0.40. ^{1}H NMR (400 MHz, $CDCl_3$) δ 8.81 (d, *J* = 6.1 Hz, 2H), 8.26 (d, *J* = 7.4 Hz, 2H), 7.52 (d, *J* = 9.2 Hz, 1H), 7.24 (s, 1H), 6.90 (s, 1H), 6.78 (dd, *J* = 9.2, 2.5 Hz, 1H), 4.42 (t, *J* = 7.4 Hz, 2H), 3.66 (q, *J* = 7.1 Hz, 4H), 3.55 (t, *J* = 6.5 Hz, 2H), 2.51 (s, 3H), 2.03 (quint, *J* = 7.4 Hz, 2H), 1.79 (quint, *J* = 6.5 Hz, 2H), 1.57 (m, 2H), 1.43 (m, 2H), 1.29 (t, *J* = 7.1 Hz, 6H). ^{13}C NMR (126 MHz, $CDCl_3$) δ 167.52, 155.66, 152.87, 152.34, 149.88, 142.58, 126.39, 121.33, 118.54, 112.16, 111.18, 110.86, 97.85, 79.23, 59.67, 45.67, 44.97, 32.22, 31.27, 26.30, 25.48, 19.15, 12.82. HRMS (ESI) calculated for $[C_{27}H_{33}ClN_3O]^+$ 450.23067, found 450.23074.

(*E*)-1-(7-Chloroheptyl)-4-(cyano(7-(diethylamino)-4-methyl-2*H*-chromen-2-ylidene)methyl)pyridin-1-ium bromide (COUPY-CA7)

1-Bromo-7-chloroheptane (496 µL, 3.02 mmol, 20 equiv.) was added to a solution of the coumarin (50 mg, 0.15 mmol, 1 equiv.) in anhydrous acetonitrile (5 mL). The solution was stirred for 16h at 60 ºC. The residue was purified by flash column chromatography (SiO_2, 0 → 10 % MeOH in CH_2Cl_2) to yield a purple solid (38 mg, 43%). R_f (SiO_2, CH_2Cl_2/MeOH 95:5) = 0.29. ^{1}H NMR (500 MHz, DMSO) δ 8.68 (d, *J* = 7.5 Hz, 2H), 8.16 (d, *J* = 7.4 Hz, 2H), 7.71 (d, *J* = 9.1 Hz, 1H), 6.97 (m, 2H), 6.92 (s, 1H), 4.42 (t, *J* = 7.2 Hz, 2H), 3.62 (t, *J* = 6.7 Hz, 2H), 3.56 (q, *J* = 7.0 Hz, 4H), 2.54 (t, 3H), 1.87 (quint, *J* = 7.2 Hz, 2H), 1.70 (quint, *J* = 6.7 Hz, 2H), 1.31 (m, 4H), 1.23 (m, 2H), 1.18 (t, *J* = 7.0 Hz, 6H). ^{13}C NMR (126 MHz, $CDCl_3$) δ 166.69, 154.82, 152.51,

151.94, 148.79, 142.90, 127.02, 120.78, 118.26, 111.81, 110.44, 110.29, 96.39, 78.03, 58.66, 45.35, 44.19, 31.87, 30.43, 27.61, 26.02, 25.32, 18.41, 12.40. HRMS (ESI) calculated for $[C_{28}H_{35}ClN_3O]^+$ 464.24632, found 464.24660.

7 NMR spectra

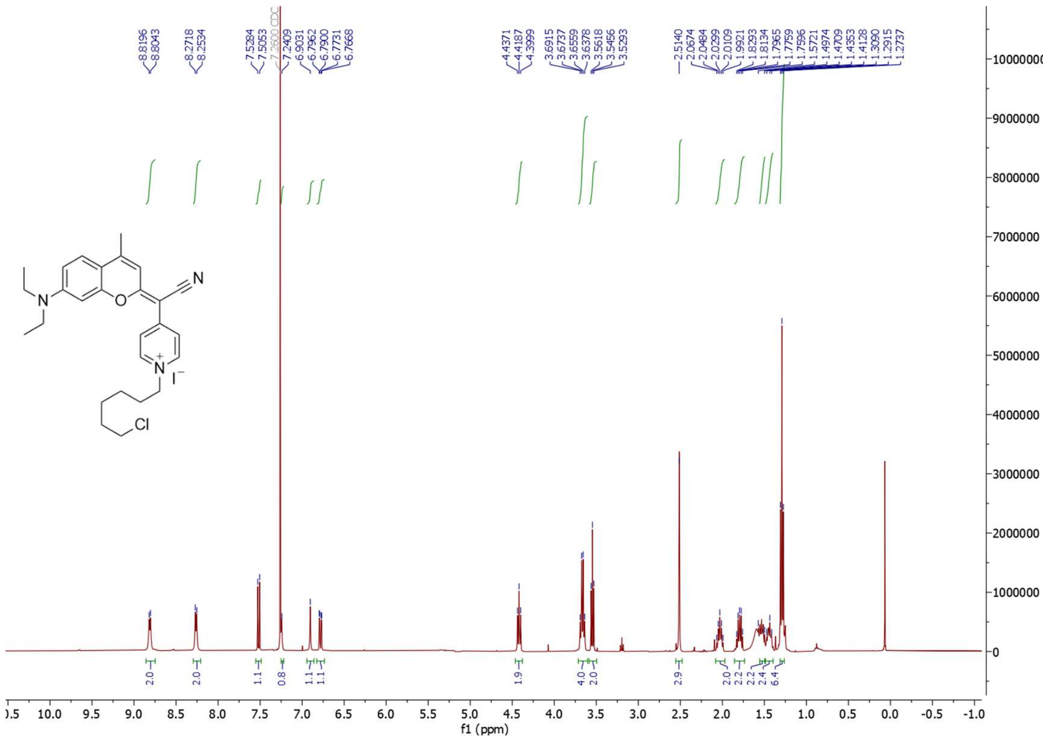

Figure S6. ^{1}H-NMR spectra of COUPY-CA6.

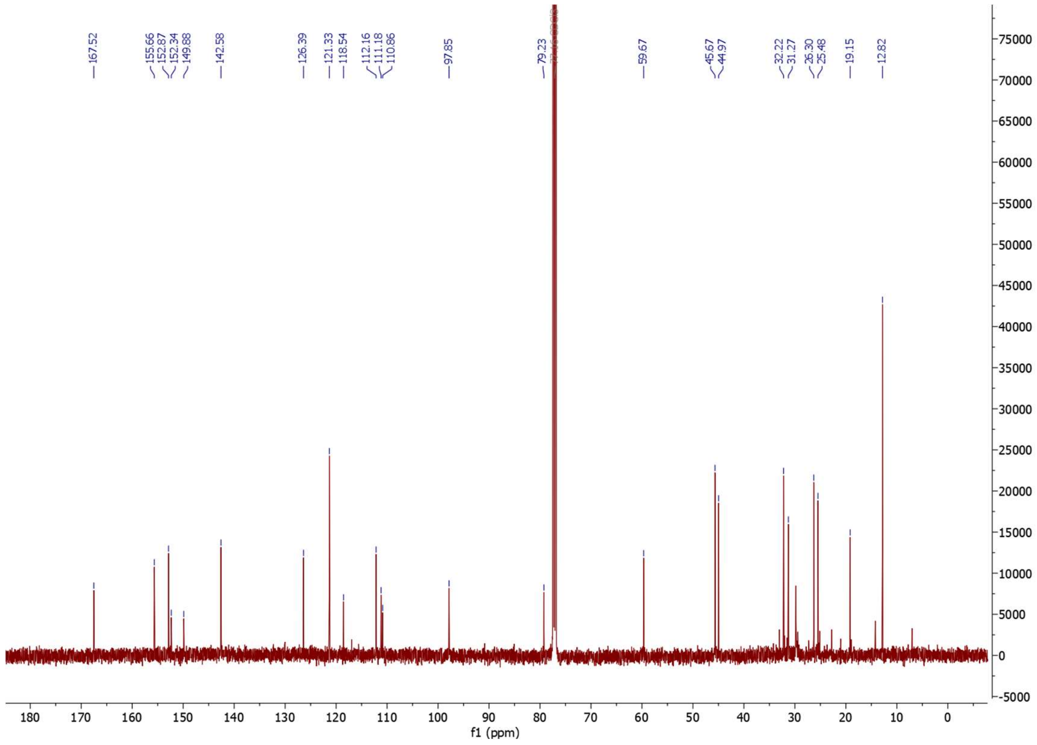

Figure S7. ^{13}C-NMR spectra of COUPY-CA6.

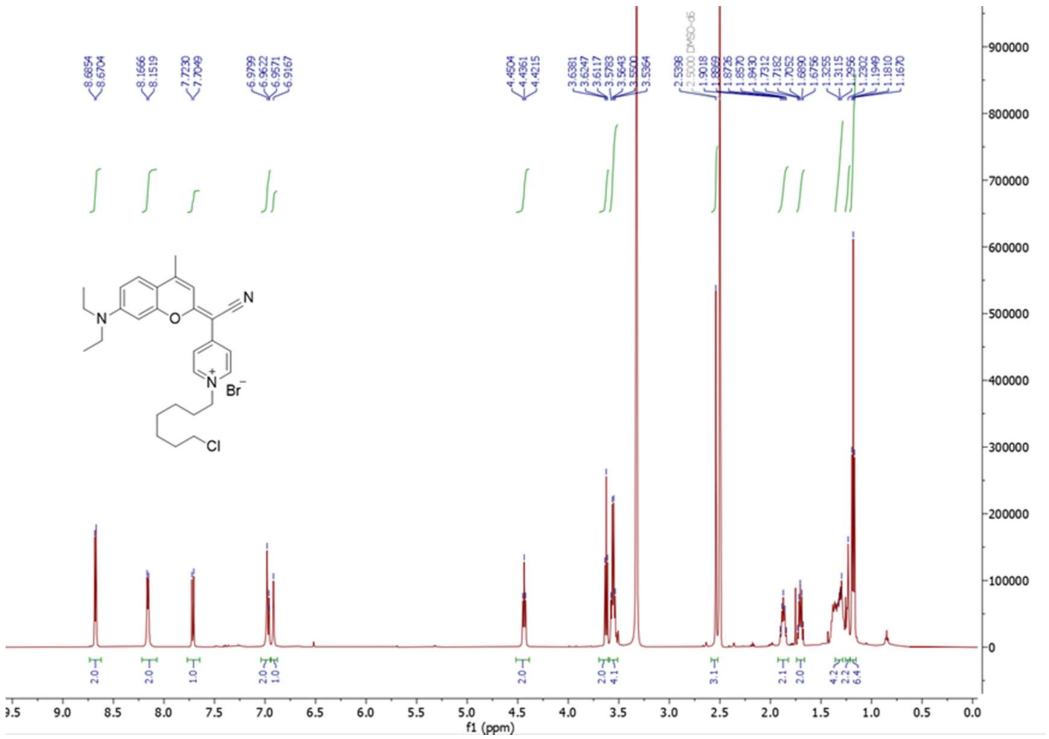

Figure S8. ^{1}H-NMR spectra of COUPY-CA7.

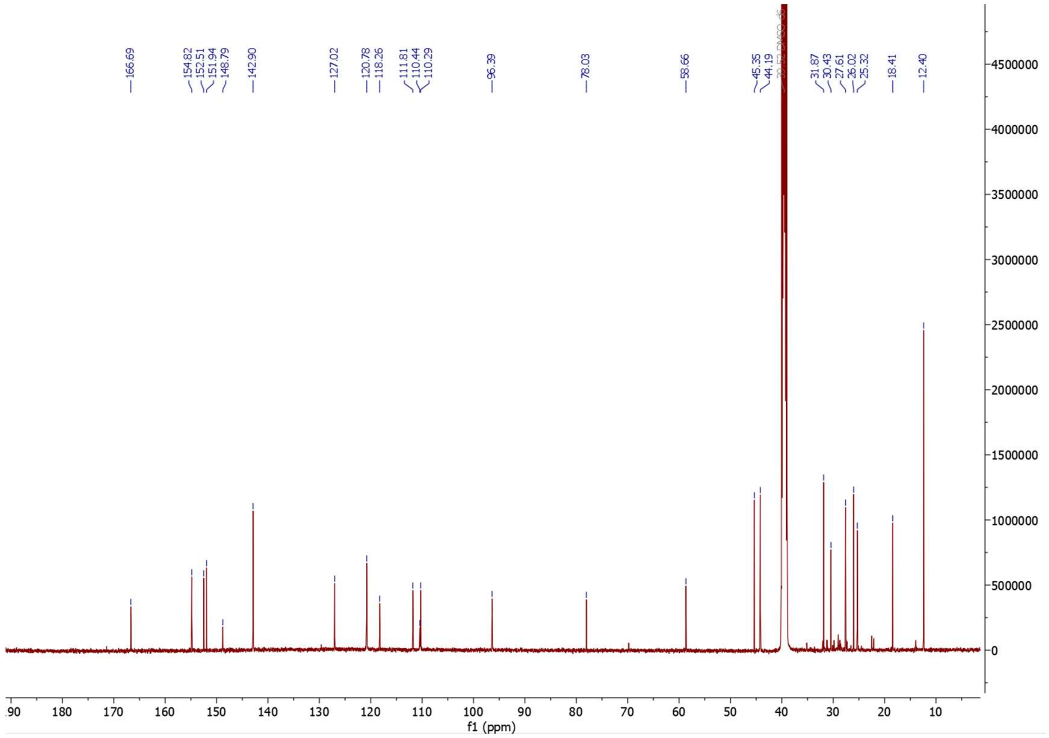

Figure S9. ^{13}C-NMR spectra of COUPY-CA7.

8 References

[1] G. R. Fulmer, A. J. M. Miller, N. H. Sherden, H. E. Gottlieb, A. Nudelman, B. M. Stoltz, J. E. Bercaw, K. I. Goldberg, *Organometallics* **2010**, *29*, 2176—2179.

[2] P. Wang, F. Hecht, G. Ossato, S. Tille, S. E. Fraser, J. A. Junge, *Biomed. Opt. Express* **2021**, *12*, 3463.

[3] J. Wohlwend, G. Corso, S. Passaro, M. Reveiz, K. Leidal, W. Swiderski, T. Portnoi, I. Chinn, J. Silterra, T. Jaakkola, R. Barzilay, **2024**, biorxiv, DOI 10.1101/2024.11.19.624167.

[4] R. Ishitani, Y. Moriwaki, **2025**, biorxiv, DOI 10.1101/2025.03.25.645362.

[5] J. V. D. S. Guerra, H. V. Ribeiro-Filho, G. E. Jara, L. O. Bortot, J. G. D. C. Pereira, P. S. Lopes-de-Oliveira, *BMC Bioinformatics* **2021**, *22*, 607.

[6] S. Mitternacht, *F1000Res* **2016**, *5*, 189.

[7] E. Jurrus, D. Engel, K. Star, K. Monson, J. Brandi, L. E. Felberg, D. H. Brookes, L. Wilson, J. Chen, K. Liles, M. Chun, P. Li, D. W. Gohara, T. Dolinsky, R. Konecny, D. R. Koes, J. E. Nielsen, T. Head□Gordon, W. Geng, R. Krasny, G. Wei, M. J. Holst, J. A. McCammon, N. A. Baker, *Prot. Sci.* **2018**, *27*, 112–128.
